# Functional Ultrasound Imaging of Spinal Cord Hemodynamic Responses to Epidural Electrical Stimulation: A Feasibility Study

**DOI:** 10.3389/fneur.2019.00279

**Published:** 2019-03-26

**Authors:** Pengfei Song, Carlos A. Cuellar, Shanshan Tang, Riazul Islam, Hai Wen, Chengwu Huang, Armando Manduca, Joshua D. Trzasko, Bruce E. Knudsen, Kendall H. Lee, Shigao Chen, Igor A. Lavrov

**Affiliations:** ^1^Department of Radiology, Mayo Clinic, Rochester, MN, United States; ^2^Department of Neurologic Surgery, Mayo Clinic, Rochester, MN, United States; ^3^Department of Physiology and Biomedical Engineering, Mayo Clinic, Rochester, MN, United States; ^4^Department of Physical Medicine and Rehabilitation, Mayo Clinic, Rochester, MN, United States; ^5^Department of Neurology, Mayo Clinic, Rochester, MN, United States; ^6^Institute of Fundamental Medicine and Biology, Kazan Federal University, Kazan, Russia

**Keywords:** functional ultrasound, spinal cord, hemodynamic responses, spinal cord injury, ultrafast imaging, electrical stimulation

## Abstract

This study presents the first implementation of functional ultrasound (fUS) imaging of the spinal cord to monitor local hemodynamic response to epidural electrical spinal cord stimulation (SCS) on two small and large animal models. SCS has been successfully applied to control chronic refractory pain and recently was evolved to alleviate motor impairment in Parkinson's disease and after spinal cord injury. At present, however, the mechanisms underlying SCS remain unclear, and current methods for monitoring SCS are limited in their capacity to provide the required sensitivity and spatiotemporal resolutions to evaluate functional changes in response to SCS. fUS is an emerging technology that has recently shown promising results in monitoring a variety of neural activities associated with the brain. Here we demonstrated the feasibility of performing fUS on two animal models during SCS. We showed *in vivo* spinal cord hemodynamic responses measured by fUS evoked by different SCS parameters. We also demonstrated that fUS has a higher sensitivity in monitoring spinal cord response than electromyography. The high spatial and temporal resolutions of fUS were demonstrated by localized measurements of hemodynamic responses at different spinal cord segments, and by reliable tracking of spinal cord responses to patterned electrical stimulations, respectively. Finally, we proposed optimized fUS imaging and post-processing methods for spinal cord. These results support feasibility of fUS imaging of the spinal cord and could pave the way for future systematic studies to investigate spinal cord functional organization and the mechanisms of spinal cord neuromodulation *in vivo*.

## Introduction

Over the last decades, epidural electrical spinal cord stimulation (SCS) was successfully implemented to help patients with chronic intractable pain (1–3). Meanwhile, SCS was reported as a promising alternative strategy to alleviate symptoms of motor impairments for multiple sclerosis (4, 5) and Parkinson's disease (6–9), and to improve motor (10–14) and autonomic functions (15) in patients with spinal cord injury. The therapeutic effects of SCS rely on the stimulation parameters used (intensity, frequency, pulse width, burst vs. continuous stimulation, electrode configuration, etc.). At the same time, the mechanisms and neural structures through which SCS inhibits chronic pain and enables motor control remain unclear, although several hypotheses were supported by computational simulations (16–18) and data, primarily obtained from electrophysiological recordings (19, 20). Electromyography (EMG) is widely used as a diagnostic tool for neuromuscular disease and a research tool for disorders of motor control. However, the EMG signal is limited and can provide one-dimensional information concerning the activation of spinal cord neurons. In this context, a combination of emerging, innovative techniques providing high spatial and temporal resolution, and electrophysiology techniques could provide critical information on mechanisms of SCS and further facilitate optimizations of SCS protocols. Spatial and/or temporal resolution of available functional imaging tools, such as PET and MEG, are far below what is required for evaluation of the spinal cord functional changes during SCS. Although the spatial resolution of functional magnetic resonance imaging (fMRI) reaches submillimeter with ultra-high magnetic field (21, 22), the size of MR machine can be prohibitive for an intraoperative monitoring.

Functional ultrasound (fUS) imaging has the potential to complement these techniques at low cost. fUS is an emerging method that leverages the novel ultrafast plane wave imaging technique and the neurovascular coupling effect to monitor hemodynamic responses of tissue associated with neural activities (23). Ultrafast plane wave imaging allows acquisition and accumulation of ultrasound data at 10–20 kHz frame rate, significantly boosting the Doppler sensitivity to small vessels for fUS imaging (24–26). The rich spatiotemporal information of ultrafast plane wave data also allows implementation of more robust and intelligent tissue clutter filters based on singular value decomposition (SVD) (27–29), further improving the sensitivity of monitoring small vessel hemodynamic responses for fUS. In contrast to fMRI which responds to both hemodynamic and metabolic variations, fUS is only sensitive to hemodynamic effects (23, 30). Therefore, interpretations of fUS results are not confounded by the complex interactions between the hemodynamic and metabolic effects (31). As compared to other imaging techniques, fUS has higher spatial and temporal resolutions and also potentially can be performed on freely moving animals with miniaturized transducer size for long-term and real-time monitoring (32, 33). This opens new directions for potential applications of fUS, since currently there is no available technique that could evaluate functional changes in spinal cord in real-time *in vivo*. fUS could help in evaluation of hemodynamic response during electrode placement in order to optimize leads location for neuromodulation therapies and for intraoperative monitoring of spinal cord hemodynamics during surgical procedures. Finally, fUS may help to generate important information about spinal cord functional organization, and particularly, could help to trace circuitry response during pharmacological interventions and neuromodulation.

One disadvantage of fUS is that ultrasound cannot effectively penetrate through the bone. Therefore, fUS typically requires removal or thinning of the skull to access the targeted tissue such as brain (23, 31). Nevertheless, fUS has demonstrated promising results in monitoring a wide range of brain activities involved with visual, auditory, olfactory, and motor functions (23, 34–36), imaging brain intrinsic connectivity (37), and measuring brain activities of humans including neonates (38) and during surgery (39). A comprehensive review of current preclinical and clinical applications of fUS was recently published in (40).

To the best of our knowledge, this is the first attempt of implementing fUS to study the effect of spinal cord stimulation in animal models. Here we present a methodology and work flow, including the optimized subpixel motion registration, SVD-based clutter filtering, and hemodynamic response quantification, to validate the feasibility of using fUS to examine the SCS response. The capability of the proposed work flow was tested on two species (rat and swine). Specific spinal cord hemodynamic responses associated with different SCS parameters were evaluated, including different voltages, and stimulation patterns.

## Materials and Methods

Experimental procedures were approved by the Mayo Clinic Institutional Animal Care and Use Committee. The National Institutes of Health Guidelines for Animal Research (Guide for the Care and Use of Laboratory Animals) were observed rigorously. Animals were kept in controlled environment (21°C, 45% humidity) on a 12-h light/dark cycle.

### Rat Study Procedure

**Figure 1:**
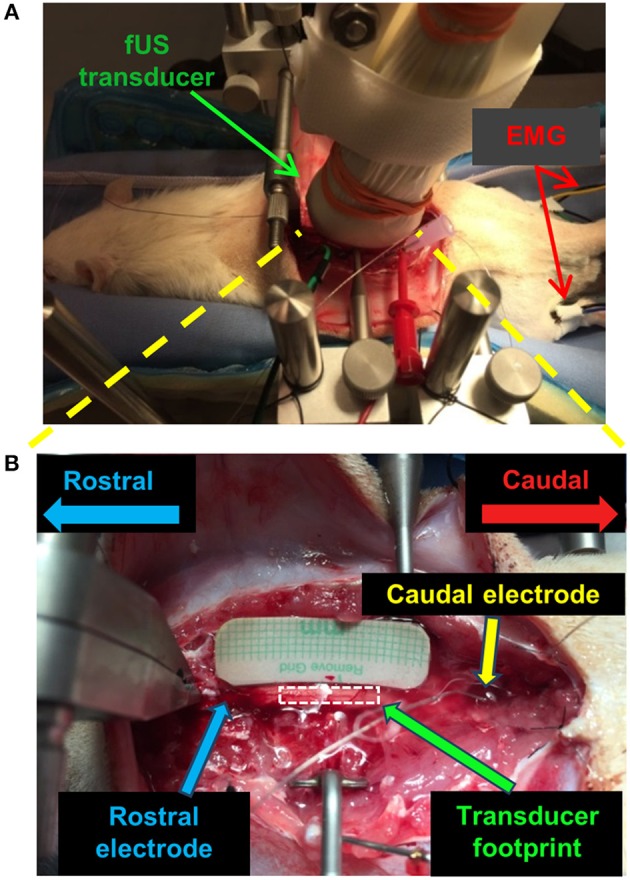
fUS imaging setup for the spinal cord stimulation study on a rat model. **(A)** Optical image of the positioning of the fUS transducer on the spinal cord. **(B)** Optical image of the targeted imaging region of the spinal cord with the fUS transducer removed. A similar setup was used for the swine study.

Sprague-Dawley rats (3 males, 325–350 gr, *ad libitum* access to water and food) were anesthetized with isoflurane (1.5–3%). Laminectomies were performed at T13-L2 and the spinal cord was exposed. Two Teflon coated stainless steel wires were placed at T13 and L2 and sutured on dura (corresponding approximately to L2 and S1 segments of the spinal cord). Small windows were opened between T11-L12 and L3-L4 allowing wires to be passed under the T12 and L3 vertebrae. A small notch (0.5 mm) facing the spinal cord was made on the Teflon coating, serving as the stimulating electrode. Breathing motion was minimized by fixing the spine using a custom-made frame composed of a clamp holding the Th12 spinous process and two pieces retracting back muscles on both sides. Additionally, two rods were secured over the coxal bones in order to hold up the pelvic girdle. Dorsal skin flaps were attached around the frame to form a pool facilitating transducer positioning (Figure 1). SCS consisted of 0.5 ms squared pulses delivered at 40 Hz in monopolar or bipolar configurations. Two reference electrodes were inserted bilaterally in back muscles. EMG signals were recorded using dual needle electrodes (Medtronic, Memphis, TN) inserted bilaterally in tibialis anterior (TA) and gastrocnemius (GAS) hind limb muscles. Warm saline solution (1.5 ml) was administered S.C. every 2 h. At the end of the experiment, animals were euthanized using pentobarbital (150 mg/kg I.P.).

### Swine Study Procedure

A domestic white swine (male, 8 weeks old, 25 kg, *ad libitum* access to water, fed once daily) was initially anesthetized using a mixture of telazol (5 mg/kg) and xylazine (2 mg/kg I.V.). Anesthesia was maintained using isoflurane (1.5–3%). For analgesia, fentanyl (2–5 mg/kg/h) was administered throughout the experiment. Similar surgical procedures as described in the previous section were performed in swine (41). Two Teflon stainless steel wires were placed onto L4 and L5-L6 and sutured on dura after laminectomies were performed at L1-L6. Back muscles were retracted and the spine stabilized using 4 blunt tip rods that attached the spine to a custom-made frame. SCS was delivered at 40 Hz, 0.5 ms pulse width in bipolar configuration. A reference electrode was inserted in the back muscles. Needle electrodes (Medtronic, Memphis, TN) were inserted bilaterally in TA and GAS hind limb muscles to monitor EMG responses during SCS. At the end of the experiment, the subject was euthanized (sodium pentobarbital 100 mg/kg I.V.).

### fUS Imaging Setup

A Verasonics Vantage ultrasound system (Verasonics Inc., Kirkland, WA) and a Verasonics high frequency linear array transducer L22-14v (Verasonics Inc., Kirkland, WA) with center frequency of 15 MHz were used in this study. Figure 1 shows the fUS imaging setup. The fUS transducer was positioned on the spinal cord between the rostral and caudal electrodes. An imaging field-of-view (FOV) was carefully selected to align with the longitudinal dimension of the spinal cord and intersect with the central canal (Figure 1B). The position of the fUS transducer was fixed throughout the study. A thin layer of mineral oil was added between the fUS transducer and the spinal cord for acoustic coupling.

**Figure 2:**
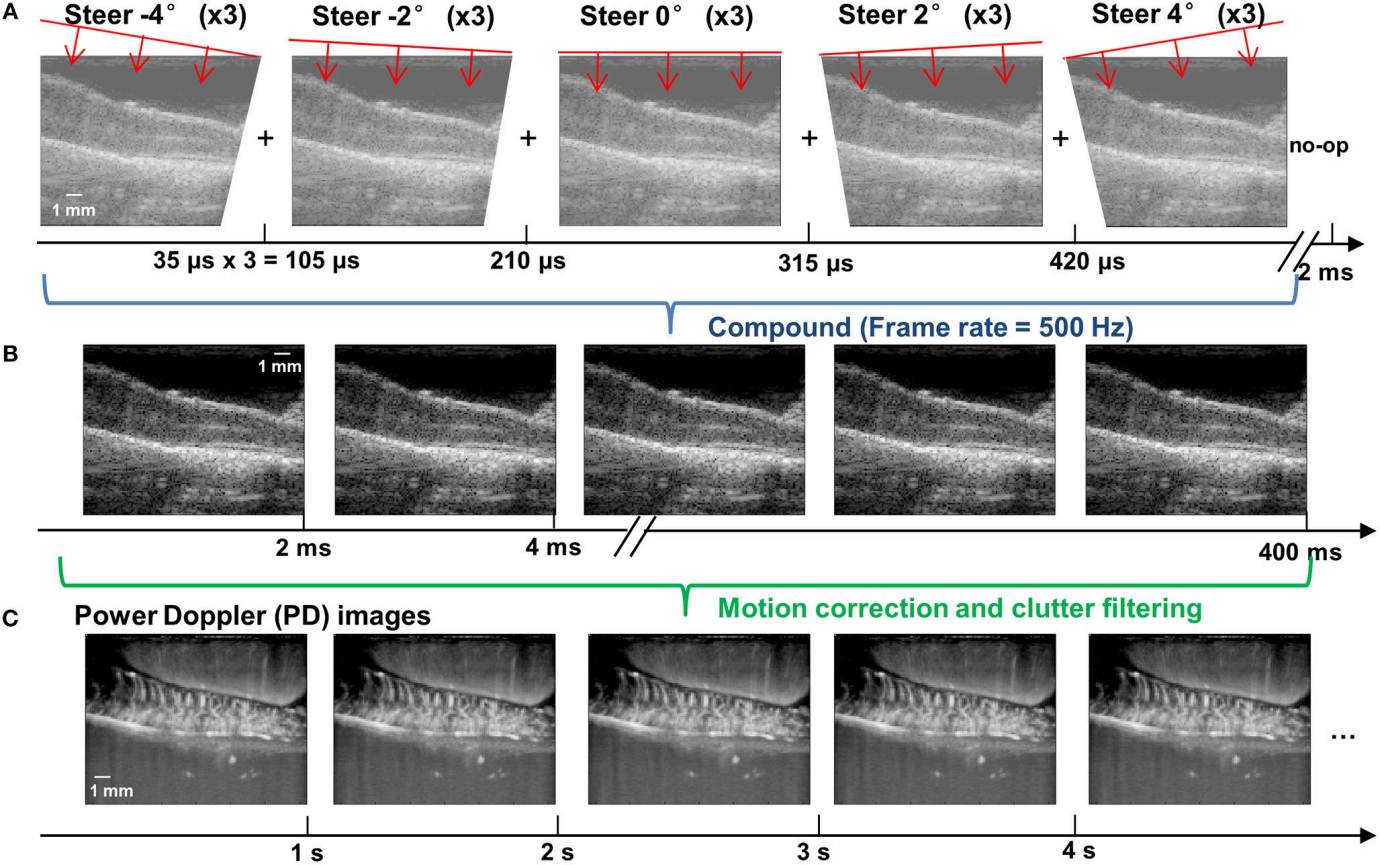
fUS imaging sequence based on ultrafast compounding plane wave imaging. **(A)** Schematic plots of the steering angles of the plane waves and the corresponding low-quality plane wave images. The time axis indicates the imaging frame rate. A no-op was added to the end of each group of compounding angles to satisfy a post-compounding frame rate of 500 Hz. **(B)** Post-compounding high-quality ultrasound data with an effective PRF of 500 Hz. Each high quality image is compounded from 15 steered plane wave images (5 angles × 3 repetitions for each angle). **(C)** Power Doppler images obtained after the motion correction and clutter filtering processing steps. Each PD image was generated from 200 Doppler ensembles (i.e., the high-quality post-compounding ultrasound data shown in **B**). The final fUS imaging frame rate was 1 Hz (that is, one PD image per second). The depth and width of the images are 9.86 and 12.8 mm, respectively.

An ultrafast compounding plane wave imaging-based fUS imaging sequence was developed for the study. As shown in Figure 2A, five steered plane waves (−4 to 4°, with 2° of step angle) were transmitted with each steering angle repeatedly transmitted three times to boost signal-to-noise-ratio (SNR). This compounding scheme has an equivalent SNR performance to a conventional 15-angle compounding sequence, but reduces the beamforming computational cost by a factor of 3 (32). The pulse repetition interval was 35 μs (corresponding to a pulse repetition frequency (PRF) of 28.6 kHz), and the total time cost for transmitting and receiving all 15 transmissions was 525 μs. To satisfy a post-compounding PRF of 500 Hz, a 1,475 μs no-op time was added to each group of compounding transmissions (Figure 2A). After coherent compounding (24), high quality ultrasound data was obtained (Figure 2B) and used as Doppler ensembles for future processing. A total of 200 Doppler ensembles (400 ms duration) were collected within each second to produce one power Doppler (PD) image per second (Figure 2C). For the rat experiment, a total of 120 s of fUS data was collected (corresponding to 24,000 frames of high frame-rate ultrasound data) for each trial of SCS, including 30 s of baseline measurement, 20 s of ES measurement, and 70 s of recovery measurement. Five trials were repeated for each SCS configuration. For the swine experiment, a total of 30 s of fUS data was collected (6,000 frames), including 5 s of baseline, 15 s of stimulation, and 10 s of recovery. Five trials were repeated for each SCS configuration.

For data synchronization with the SCS and EMG measurements, the Verasonics system was programmed to send a trigger-out signal at the beginning of each second when the first steered plane wave was transmitted. The trigger-out signal was recorded together with the SCS and EMG signals for post-processing.

### fUS Post-processing Steps

#### Motion Correction

To facilitate accurate fUS measurements of hemodynamic responses, we developed a robust and fast sub-pixel motion correction algorithm to remove tissue motion induced by breathing and SCS. Motion correction was applied both on the original high frame-rate ultrasound data before clutter filtering (e.g., Figure 2B), and on the PD images after clutter filtering (e.g., Figure 2C). The motion correction method was based on the principles of phase correlation-based sub-pixel registration introduced in (42). Briefly, the method by Foroosh et al. (42) derived an analytical solution of the phase correlation function between images that are shifted by non-integer number of pixels (Δ*x*, Δ*z*), and presented a method of using the main peak and side peaks of the inverse Fourier transform of the phase correlation function (*C*) to calculate the sub-pixel displacement:

$$\begin{array}{r} \Delta x=\frac{C\left(1,0\right)}{C\left(1,0\right)\pm C\left(0,0\right)} \end{array}$$

(1) $$\begin{array}{r} \Delta z=\frac{C\left(0,1\right)}{C\left(0,1\right)\pm C\left(0,0\right)} \end{array}$$

where *C*(0,0) indicates the main peak (i.e., location of the pixel with highest positive pixel value) and *C*(1,0) and *C*(0,1) indicates the side peaks (i.e., location of the pixel with second highest positive pixel value) along *x*-dimension and *z*-dimension, respectively. To improve the robustness of Equation (1) for ultrasound applications, we added additional measurements of Δ*x*′ and Δ*z*′ using the main peak and side peaks with highest negative pixel value:

$$\begin{array}{r} \Delta x^{\prime }=\frac{C\left(-1,0\right)}{-C\left(-1,0\right)\pm C\left(0,0\right)} \end{array}$$

(2) $$\begin{array}{r} \Delta z^{\prime }=\frac{C\left(0,-1\right)}{-C\left(0,-1\right)\pm C\left(0,0\right)} \end{array}$$

Then an average sub-pixel displacement was calculated using the results from Equations (1) and (2). Other available sub-pixel motion estimation algorithm, such as the one presented in (43) and the normxcorr2.m function in MATLAB, require heavy up-sampling of ultrasound signals in order to measure the sub-pixel motion between frames. In fUS imaging, this up-sampling procedure is extremely computationally expensive due to the large amount of ultrasound data acquired in temporal dimension. In contrast, the sub-pixel motion estimation algorithm used in this study does not require up-sampling and involves Fourier transform, which can be executed at extremely fast speed. Therefore, the computational cost can be greatly reduced with the method used in this study.

**Figure 3:**
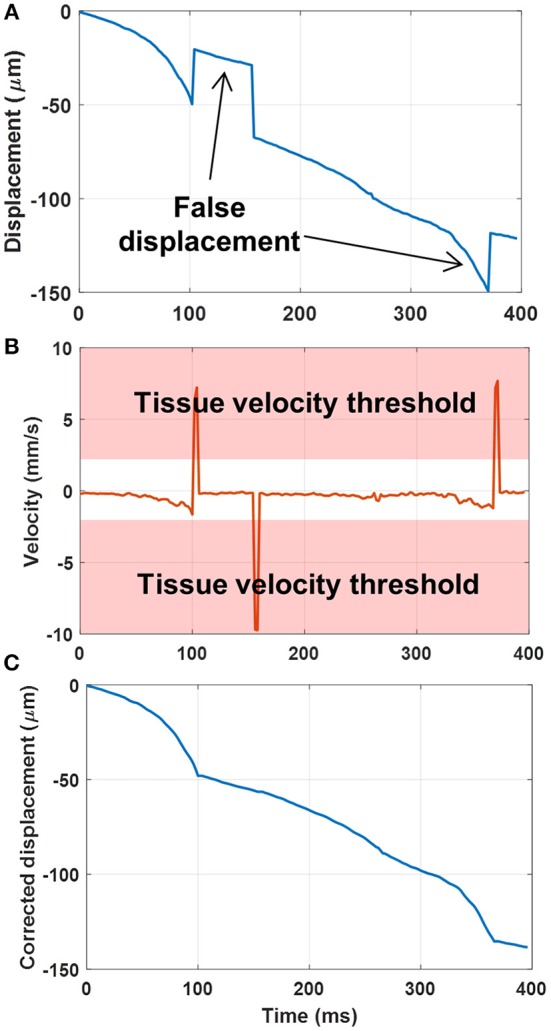
**(A)** Original displacement curve with false displacement calculations. **(B)** Taking the derivative (i.e., velocity) of the displacement curve, and applying a tissue velocity threshold. **(C)** Integral of the velocity curve after rejection of large tissue velocities to remove the false displacement calculations.

To further improve the robustness of sub-pixel displacement estimation and suppress false calculations, as shown in Figure 3, a tissue velocity curve (Figure 3B) was first derived by taking a derivative of the original displacement curve (Figure 3A). Then a tissue velocity thresholding (cutoff was determined empirically as 2 mm/s for this study) was applied to the velocity curve to reject high speed values, followed by an integral calculation to recover the displacement curve (Figure 3C). False displacement could be effectively removed by this process. This additional step was only applied to the original high frame-rate ultrasound data, not to the PD images.

Finally, to avoid creating the streaking artifacts associated with applying a phase-shift to the Fourier spectrum (due to bandlimited data), the gridded data interpolation (e.g., “griddedInterpolant.m” function in Matlab) was used to register the moved ultrasound frames.

#### Tissue Clutter Filtering

**Figure 4:**
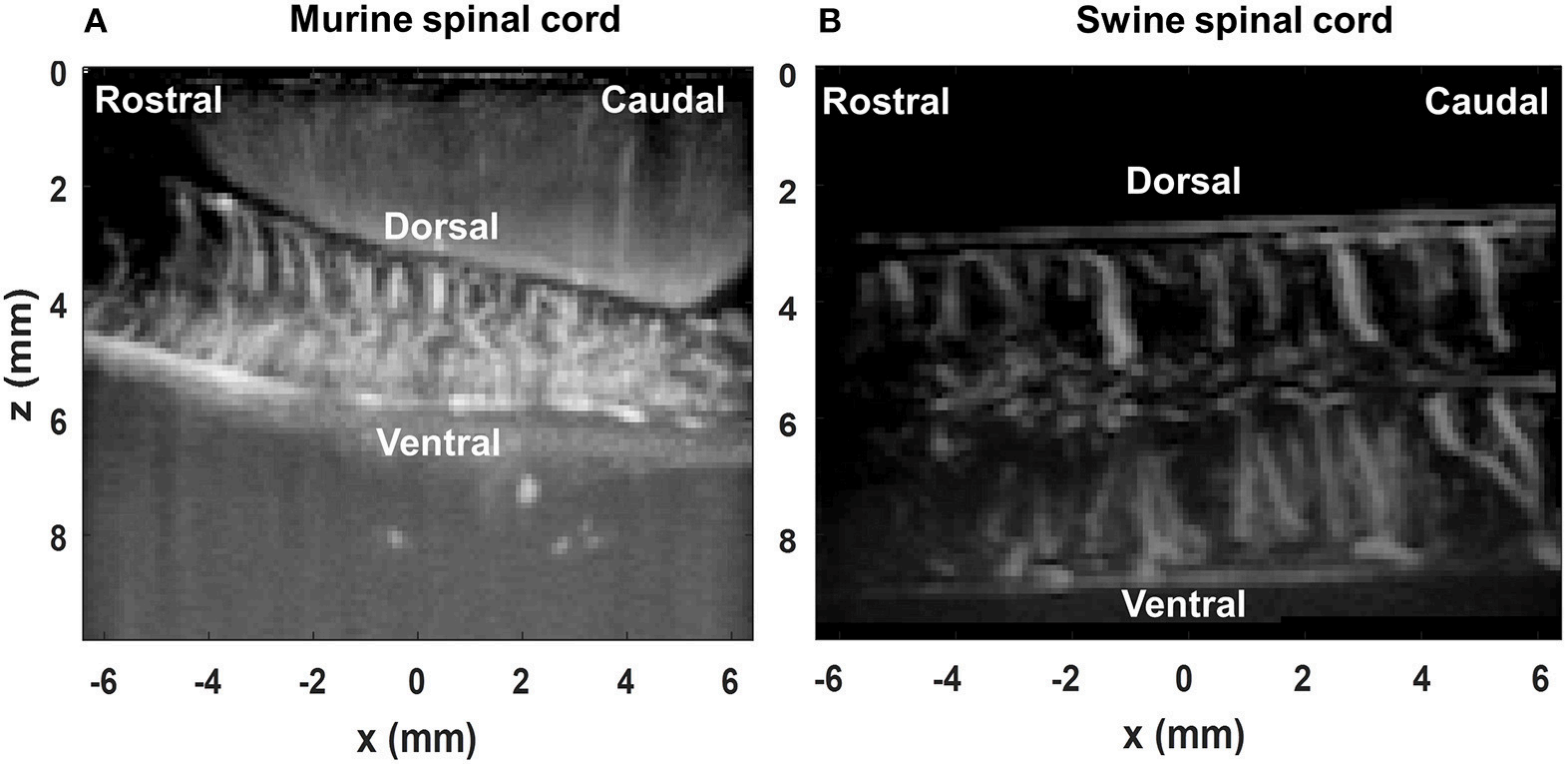
Power Doppler (PD) images of the rat spinal cord **(A)** and the swine spinal cord **(B)** post SVD clutter filtering.

The spatiotemporal SVD-based ultrasound clutter filter was used in this study to suppress tissue clutter and extract micro-vessel signals (27–29). Here we used the combination of an accelerated SVD method (44) and a noise equalization technique (45) for tissue clutter filtering. For the first 200 ultrasound ensembles in each trial, a full SVD was calculated to determine a low-cutoff singular value threshold for tissue rejection (28) and derive a noise field for noise equalization (45). The same low-cutoff value and noise field were used for the rest of the ultrasound data in the trial. Figure 4 shows the PD images after the motion correction and the clutter filtering process for the rat spinal cord (Figure 4A) and the swine spinal cord (Figure 4B).

#### Spinal Cord Hemodynamic Response Calculation and Measurement

Ultrasound Power Doppler signal measures the backscattering power of the moving blood, which reflects the blood volume at the interrogated location (e.g., each imaging pixel) (46). Here we define the spinal cord blood volume change (Δ*SCBV*) as the percentage of power Doppler (*PD*) signal variation compared to the baseline:

$$\begin{array}{l} \Delta \text{SCBV}=\frac{{\text{PD}}_{\text{stim}}-{\text{PD}}_{\text{baseline}}}{{\text{PD}}_{\text{baseline}}}\times 100\% \end{array}$$

**Figure 5:**
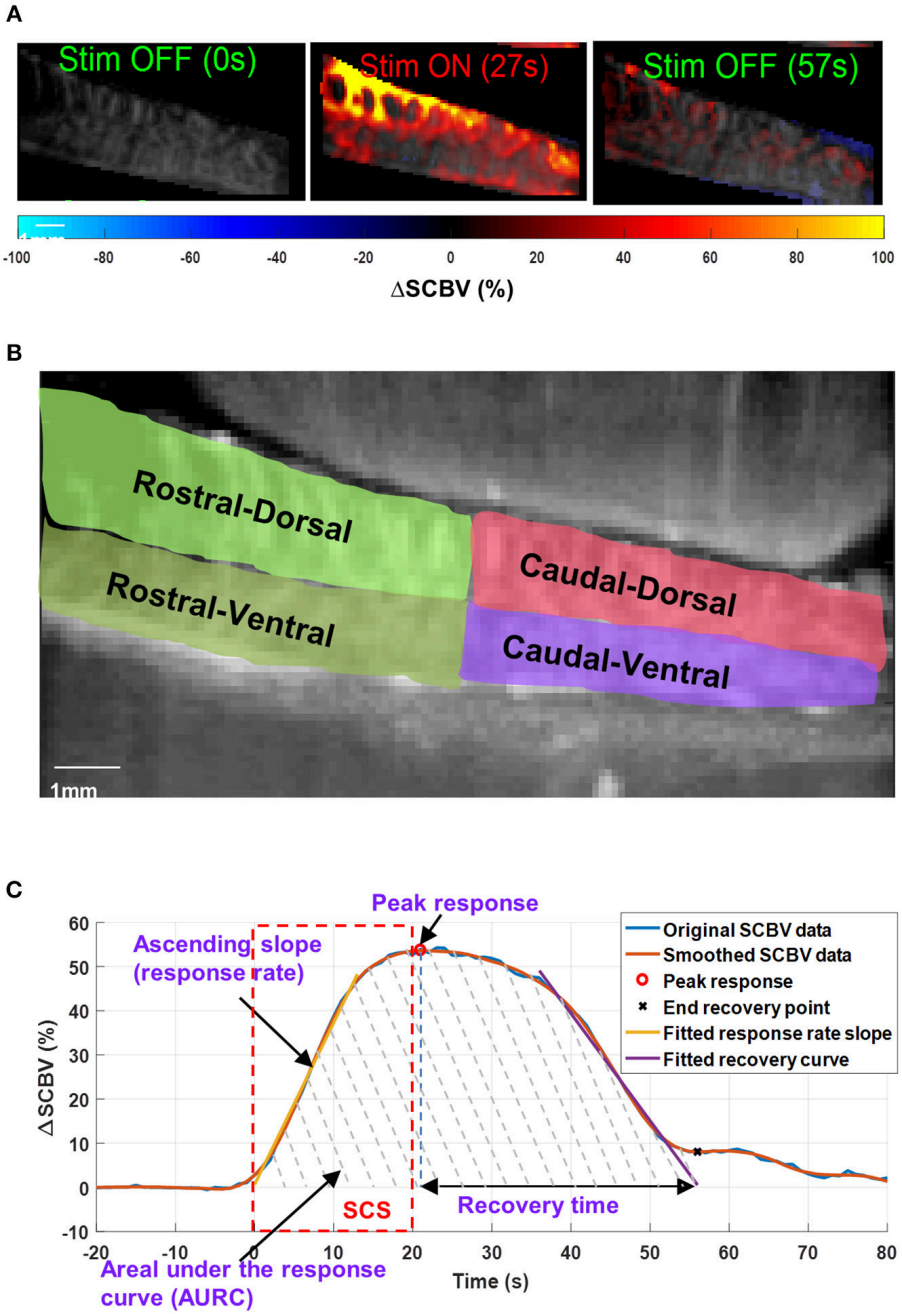
**(A)** Spinal cord hemodynamic response maps during SCS. Color map indicates the spinal cord blood volume change (Δ*SCBV*). A movie of the SCS response is provided in Supplemental Video 1. **(B)** Selection of regions-of-interest (ROIs) for local Δ*SCBV* assessment. **(C)** Indications of quantitative SCBV measurements derived for SCS response.

A Savitzky-Golay smoothing filter (47) (window length = 11, order = 1) was applied to the Δ*SCBV* measurement for each imaging pixel along the temporal direction to remove noise. Δ*SCBV* measurements with amplitude smaller than twice the standard deviation of the baseline fluctuations were rejected. The remaining Δ*SCBV* measurements were color-coded and superimposed on the PD images (Figure 5A, Supplemental Videos 1, 2 for spinal cord hemodynamic response with and without SCS).

For quantitative local Δ*SCBV* measurements, four regions-of-interest (ROIs) were selected for the rostral-dorsal, rostral-ventral, caudal-dorsal, and caudal-ventral sections of the spinal cord (Figure 5B). For each section, the average Δ*SCBV* was calculated using all pixels inside the ROI for each time point. Then the five Δ*SCBV* curves from the five repeated SCS trials were averaged and smoothed (by Savitzky-Golay filter with 5th order and 21-sample window length) for quantitative measurements, as indicated by the blue and the orange curve in Figure 5C, respectively. Four parameters including the peak response, ascending slope of the response curve (i.e., response rate), area under the response curve (AURC), and the recovery time were derived from the Δ*SCBV* curve. For the response rate, a linear fitting was performed on the ascending portion of the Δ*SCBV* curve to calculate the slope (indicated by the yellow curve in Figure 5C). To determine the end point of the SCS response and spinal cord recovery, a linear fitting was performed on the descending portion of the Δ*SCBV* curve, and the point where the fitted line intersects with the zero Δ*SCBV* axis was used as the end recovery point (indicated by the cross sign in Figure 5C). The time interval between peak response and end recovery point was calculated as the recovery time. Finally, the total area under the curve between the onset of SCS and the end recovery point was calculated as AURC, which reflects the total spinal blood volume variations within the imaging FOV in response to SCS.

## Results

### Effect of SCS on Spinal Cord Hemodynamic Change vs. Muscle Neuro Electrophysiological Change

**Figure 6:**
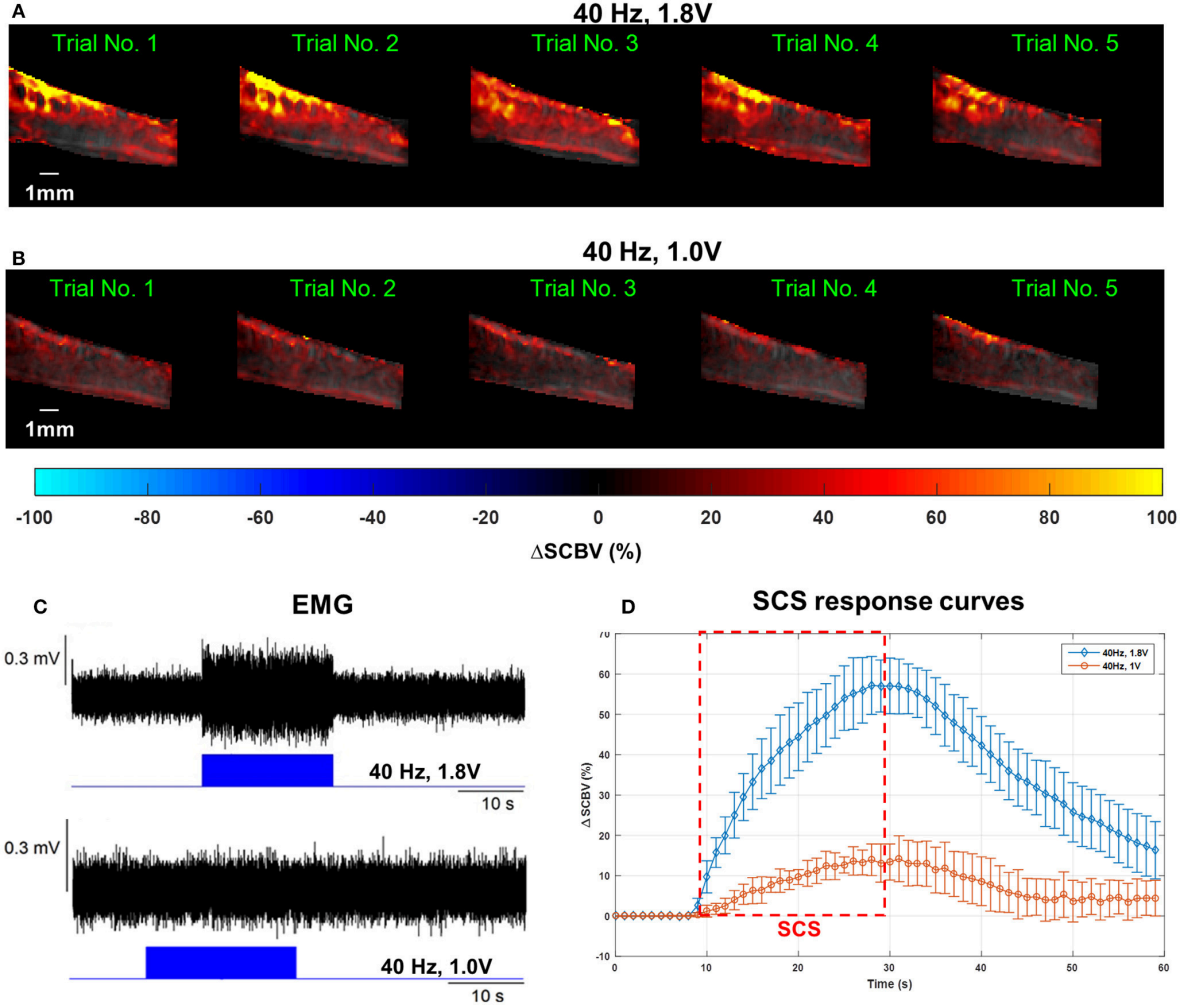
**(A–C)** Spinal cord hemodynamic response maps **(A,B)** and corresponding EMG recordings **(C)** from the GAS muscle at different SCS voltages. Corresponding fUS movies of the SCS response were provided in Supplemental Videos 2, 3, respectively. **(D)** Mean spinal cord response (dorsal) curves from different SCS voltages averaged from five trials. The error bars indicate standard deviation.

**Figure 7:**
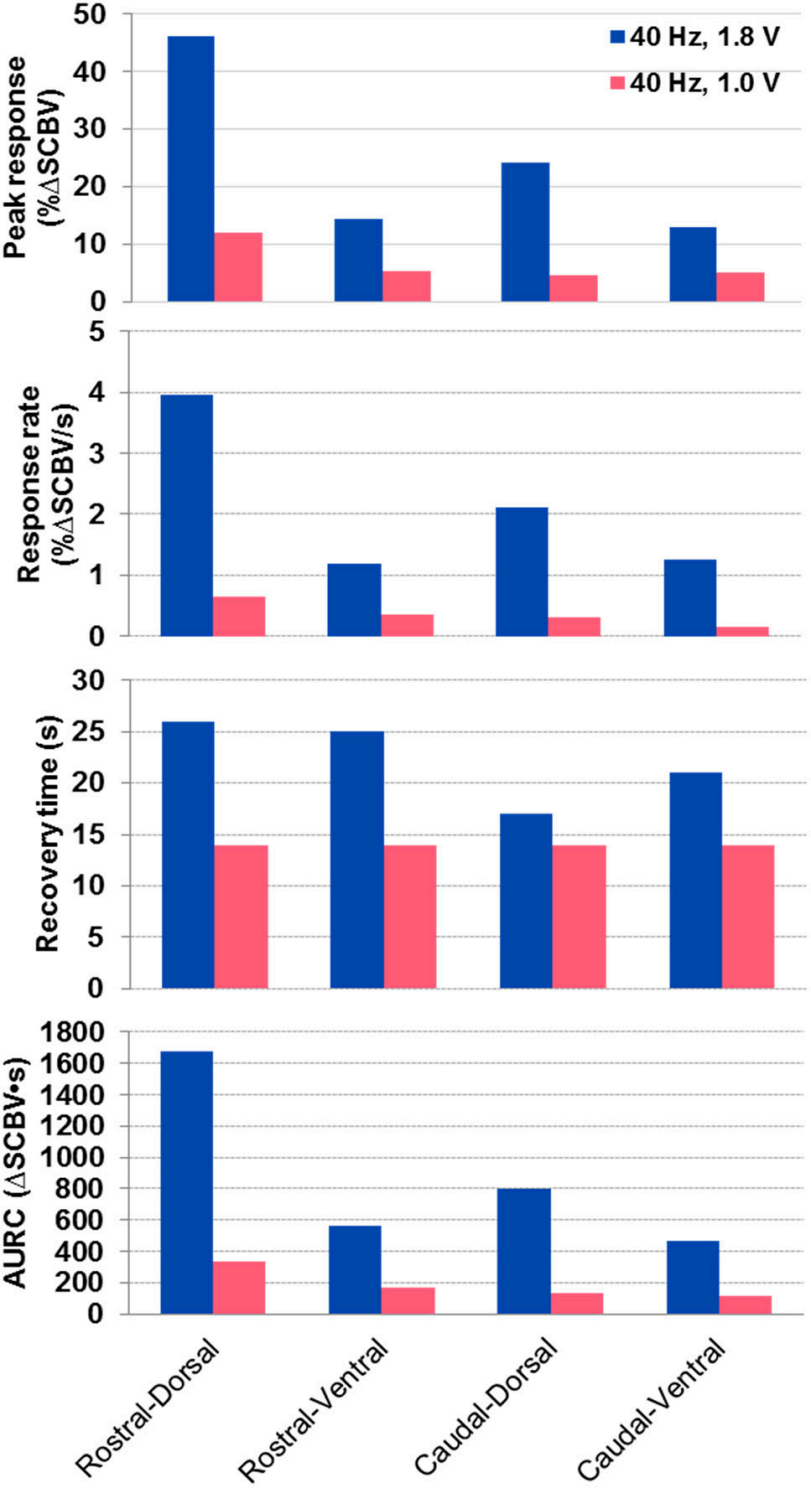
Quantitative spinal cord hemodynamic response measurements with two different SCS voltages. Measurements were obtained from averaged SCS response curves from 5 trials using the method indicated in Figure 5C. AURC, Area under the response curve.

Figure 6 shows the spinal cord hemodynamic responses to SCS on a rat model (rat #1) with different stimulation voltages (1.8 and 1.0 V) at 40 Hz SCS frequency. SCS at 1.8 V produced a clear EMG response reflected in the hemodynamic response maps and response curve (Figures 6A,C,D, and Supplemental Video 3). On the other hand, 1.0 V SCS did not produce a visible EMG response and only a weak response curve was observed primarily in dorsal part of the spinal cord (Figures 6B–D, and Supplemental Video 4). From these results, one can clearly see that higher SCS voltages produced stronger spinal cord hemodynamic responses. Figure 7 shows that all quantitative spinal cord response measurements at different sections were decreased with stimulation at lower voltage. At the same time, for both 1.8 and 1.0 V of stimulation hemodynamic changes were higher at the dorsal compared to the ventral part of the spinal cord. Increasing SCS voltage also increased hemodynamic responses in the ventral parts of the spinal cord across different segments, which correlates with the EMG observations in Figure 6C.

A gradually increased SCS voltage, from 0.4 to 1.2 V, was applied to another rat (rat #3). Supplemental Figure 1 shows the monotonic and linear relationship between the measured Δ*SCBV* and Δ*EMG* at different SCS voltages. Δ*EMG* denotes the increase in root-mean-square (RMS) of EMG signal during stimulation compared to its baseline. In our experiments we observed that different rats had different tolerance and reaction threshold to electrical stimulation. Even for the same rat, the reaction threshold could also vary with different stimulation frequency and electrode configuration. Results presented in Supplemental Figure 1 was collected from a different rat to the results in Figure 6, therefore distinct voltages were used.

### Spatial Analysis of SCS Evoked Spinal Cord Hemodynamic Response

**Figure 8:**
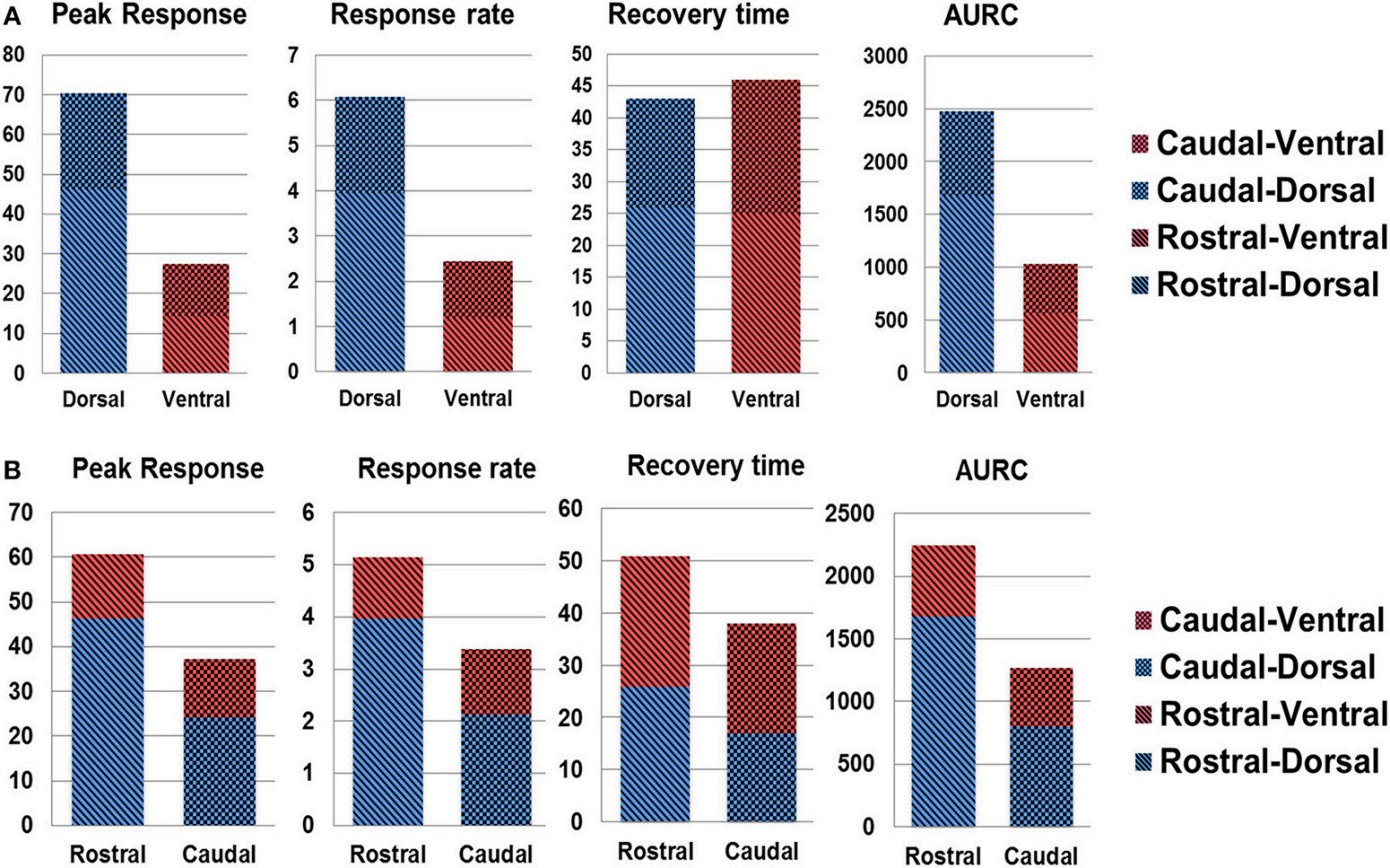
Spatial analysis of spinal cord hemodynamic response. **(A)** dorsal vs. ventral SCS response; **(B)** rostral vs. caudal response. AURC, Area under the response curve.

Figure 8 shows the quantitative spinal cord hemodynamic responses to SCS categorized by different sections of the spinal cord. The main difference in hemodynamic changes with SCS was found between activation of the dorsal and ventral part of the spinal cord with higher activity in the dorsal part across all tested segments. The difference between rostral and caudal hemodynamics was less prominent, with higher hemodynamic response on rostral segments (where the electrode was placed). These results are in agreement with observations in Figures 6A,B, where the rostral-dorsal section of the spinal cord had the highest blood volume increase during the stimulation.

### Spinal Cord Hemodynamic Response to Patterned SCS

**Figure 9:**
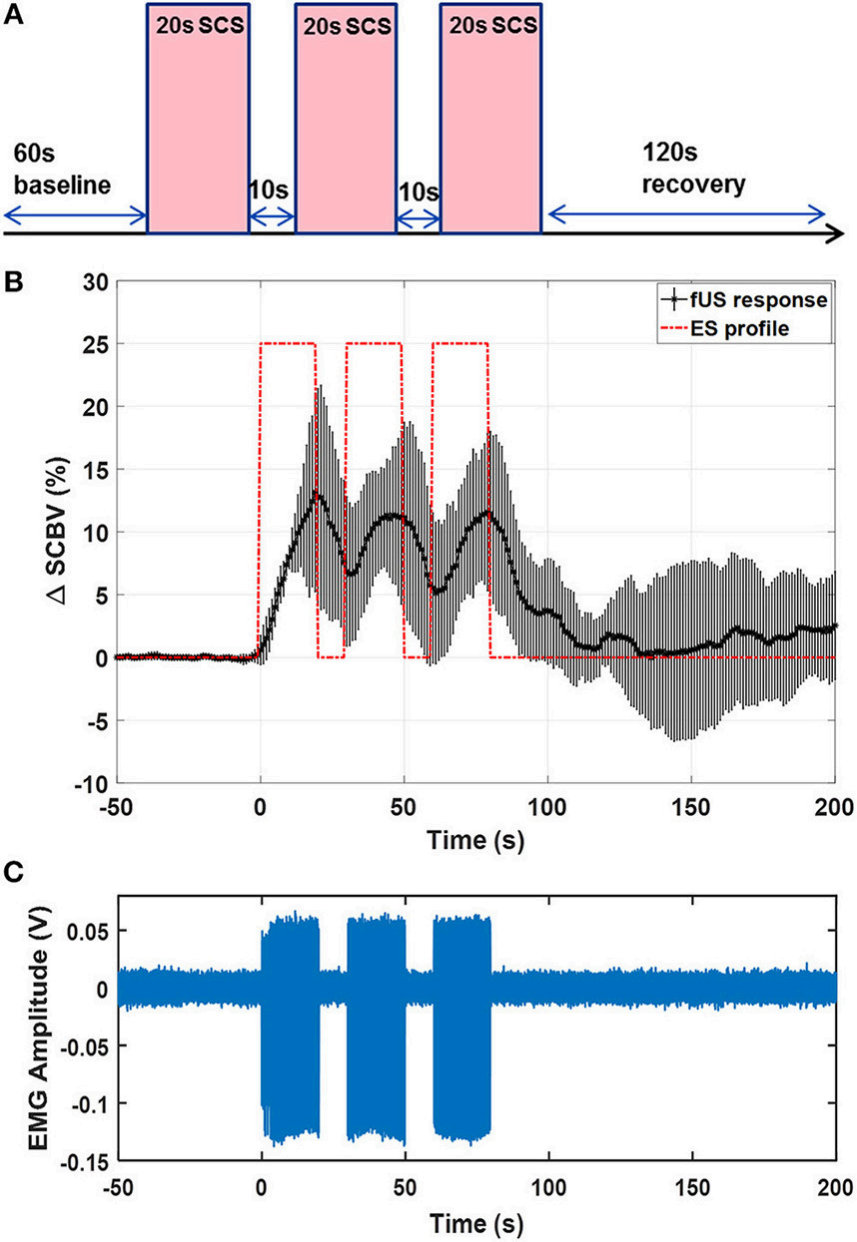
**(A)** Schematic plot of the patterned SCS. **(B)** fUS monitored spinal cord response averaged from 5 trials. Error bar indicates standard deviation. **(C)** EMG recording from the GAS muscle. The fUS response movie can be found in Supplemental Video 4.

Figure 9 shows the results of fUS monitoring of spinal cord response under a patterned SCS (rat #2). The patterned SCS consists of three ON-OFF SCS cycles, with each cycle containing a 20-s ON period and a 10-s OFF period with the SCS frequency 40 Hz and amplitude 0.6 V in bipolar configuration (Figure 9A). Compared to the result in Figure 6, a lowered stimulation voltage was used here, as the motor response threshold was different among animals and with varied SCS parameters and electrode configurations. From Figure 9B, one can clearly see the variations of spinal cord blood volume following the ON-OFF pattern of SCS. Inadequate recovery time was given between consecutive SCS periods, and consequently the spinal cord blood volume could not return to baseline value until the patterned SCS was OFF. Simultaneous EMG response is shown in Figure 9C. Supplemental Video 5 shows one representative movie of the patterned SCS response in a rat model.

### Feasibility Study on Swine Model

**Figure 10:**
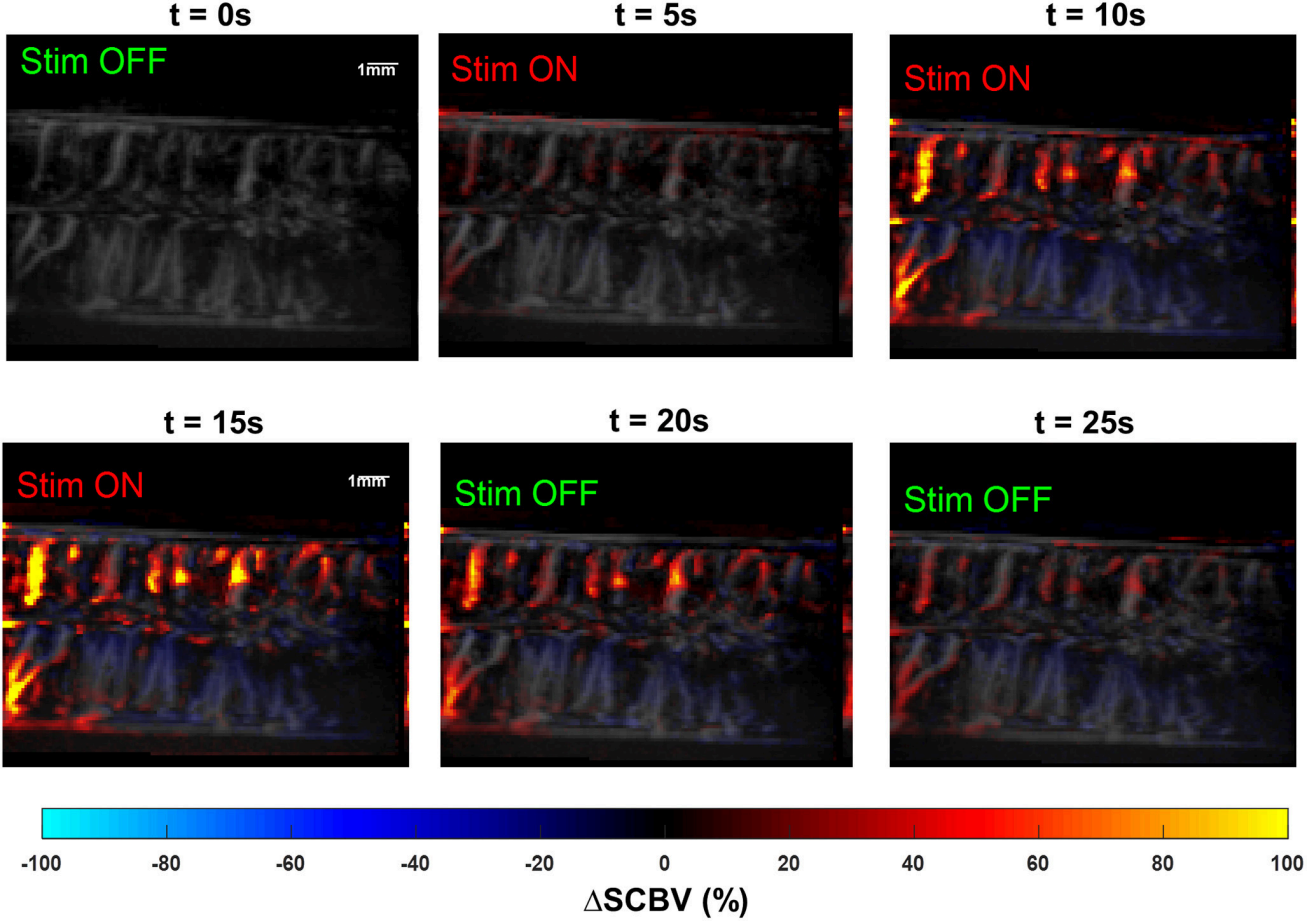
Snapshots of the fUS movie of the swine spinal cord response to SCS. The movie is provided in Supplemental Video 5.

Figure 10 shows the results of the effect of SCS on hemodynamic changes in the swine spinal cord. A 40 Hz bipolar stimulation was used with a stimulation voltage of 10 V. Higher stimulation voltage was used in the swine model compared to the rat model due to differences in SCS thresholds for these two species. Supplemental Video 6 shows the movie of the swine spinal cord response. Similar to the results observed in the rat study, the swine spinal cord showed well-correlated hemodynamic responses to the SCS. As shown in Figure 10 and Supplemental Video 6, similar to the rat study, the dorsal spinal cord had significantly higher blood volume increase than the ventral spinal cord.

## Discussion

An optimized work flow of using fUS to map local spinal cord hemodynamic response during epidural electrical stimulation was presented in this article. The proposed methodology was applied on two animal species for feasibility and capability validation. Although not a systematic study, the preliminary results presented here demonstrated great potential of fUS in monitoring and evaluating the spinal cord's hemodynamic response during epidural electrical stimulation *in vivo*.

In order to save the computational cost associated with motion correction, the sub-pixel motion registration algorithm was used in this study. This fast algorithm cannot correct for non-rigid tissue motion which may occur in *in vivo* studies. This may result in residual tissue motion that may cause false spinal cord response measurements which produces fluctuations of fUS-measured spinal cord response.

In this study, we investigated the spinal cord hemodynamic response which was compared with electrophysiological measurements during spinal cord epidural stimulation. Compared to other functional imaging techniques, fUS provides superior spatiotemporal resolutions that allow investigation of local spinal cord responses even in small models like rat and monitoring the time-varying spinal cord responses evoked by SCS. Our data also suggest that fUS is a more sensitive technique than commonly used electrophysiological assessment such as EMG and can evaluate subthreshold to motor response level of SCS.

The main objective of this study was to test the feasibility and capability of using fUS to examine the epidural stimulation evoked specific changes in spinal cord hemodynamics, measured in the lumbosacral spinal cord segments. During *in vivo* experiments in small (rat) and large (swine) animal models, epidural stimulation produced significant blood volume changes in spinal cord with clear specificity to the different areas of the spinal cord. Specific anatomical organization of the spinal cord vasculature with anterior and posterior spinal arteries divides the spinal cord into two areas, providing relatively independent blood supply for ventral and dorsal parts of the spinal cord (48–51). This difference between dorsal and ventral parts, although evident from anatomical studies, to our knowledge has not been correlated with the functional organization of the spinal cord until now. Comparison between right and left side of the spinal cord (rostral vs. caudal regions) was also important to assess the level of asymmetry in activation of spinal cord afferents, which could be functional or related to anatomical position of the electrode on the spinal cord.

In order to provide good control over the position of the fUS transducer and to reduce motion artifacts, this study was conducted on anesthetized animals. Accordingly, our current findings cannot reflect the full spectrum of spinal cord responses that can be observed in awake animals. For example, isoflurane anesthesia, used in this study, could affect vascular response by causing vasodilation (52).

One limitation of fUS is the motion artifacts induced by physiologic activities such as breathing and movement, which could affect data collection and may require sophisticated stabilization of the vertebral column and mechanical isolation from the muscles. Another limitation is direct placement of the fUS transducer on the spinal cord, since ultrasound cannot penetrate the vertebra, which is an obstacle for this technique in clinical translation. However, non-invasive fUS with microbubble-enhanced Power Doppler technique has been reported recently (40, 53), where fUS could be performed with intact skull bone. This non-invasive form of fUS imaging can be adopted and evaluated for spinal cord imaging in the future. Also, this limitation of removing vertebra could be potentially solved with miniaturization of the devices and development of implantable transducers.

Current information on spinal cord functional organization is primarily comes from electrophysiology experiments with intracellular or extracellular recordings or based on activity recorded in selected nerves or muscles. Using these approaches previous studies showed that spinal circuitry is highly sensitive to different modalities of afferent information, which determines immediate and long-term changes and complex mechanisms such as plasticity and neuroregeneration (54–56). Studies performed on acute decerebrated cats (57) suggest that epidural vs. intraspinal stimulation can activate different spinal cord networks with important role of sensory information in their modulation. The extensive convergence of afferent information on different types of neurons produces significant limitations in understanding of spinal circuitry organization with available electrophysiological tools in real-time (58, 59). Evaluation of spinal cord hemodynamic changes with fUS is a novel and highly sensitive tool that could help to provide information about real-time spinal cord activity across multiple segments and improve our understanding the spinal cord functional organization *in vivo*. As a proof-of-concept work, this study was only performed on a small and a large animal model. Massive and thorough investigations will be conducted in the future to explore the potentials of clinical translation.

## Conclusions

The importance of understanding the physiological and pathological mechanisms of the spinal cord hemodynamic regulation is critical for diagnostics, for clinical monitoring, and for developing novel therapies and new rehabilitation protocols. The results of the present study indicate that epidural stimulation can cause spinal hemodynamic changes related to complex neuronal activity of spinal circuitry in both small and large animal models. This study presents the first implementation of fUS to explore functional organization of the spinal cord hemodynamics and provides results on correlations between SCS induced neural activities and local hemodynamics changes. The fUS measurements indicate temporal and spatial resolutions not achievable by other electrophysiological methods. Future studies on modulation of neuronal activity and hemodynamic response with spinal cord stimulation will help to address critical questions about spinal cord functional organization in intact spinal cord and its acute and chronic changes related to different pathological conditions.

## Data Availability

All datasets generated for this study are included in the manuscript and/or the Supplementary Files.

## Author Contributions

PS, CC, SC, RI, KL, and IL designed the experiment. PS, CC, ST, RI, and IL drafted the manuscript. PS, CC, RI, and CH collected experiment data. PS, ST, AM, and JT wrote the algorithms for data processing. CC, RI, HW, and BK conducted the animal surgeries. All authors reviewed and participated in editing the manuscript.

### Conflict of Interest Statement

The authors declare that the research was conducted in the absence of any commercial or financial relationships that could be construed as a potential conflict of interest.

## Supplementary Material

Supplementary Figure 1 Spinal cord hemodynamic response and EMG to a gradient voltage.

Supplementary Video 1 Five trials of spinal cord hemodynamic response to electrical stimulation on a rat model.

Supplementary Video 2 Silent spinal cord hemodynamic response to an OFF electrical stimulation on a rat model.

Supplementary Video 3 Spinal cord hemodynamic response to a 40 Hz, 1.8 V, Monopolar electrical stimulation on a rat model.

Supplementary Video 4 Spinal cord hemodynamic response to a 40 Hz, 1.0 V, Monopolar electrical stimulation on a rat model.

Supplementary Video 5 Spinal cord hemodynamic response to a patterned electrical stimulation on a rat model.

Supplementary Video 6 Spinal cord hemodynamic response to a bipolar electrical simulation on a swine model.
