# Supplementary figures and images for: Functional Ultrasound Imaging of Spinal Cord Hemodynamic Responses to Epidural Electrical Stimulation: A Feasibility Study
Source: Front Neurol. 2019 Mar 26;10:279. doi: 10.3389/fneur.2019.00279 (PMC6445046; doi:10.3389/fneur.2019.00279)

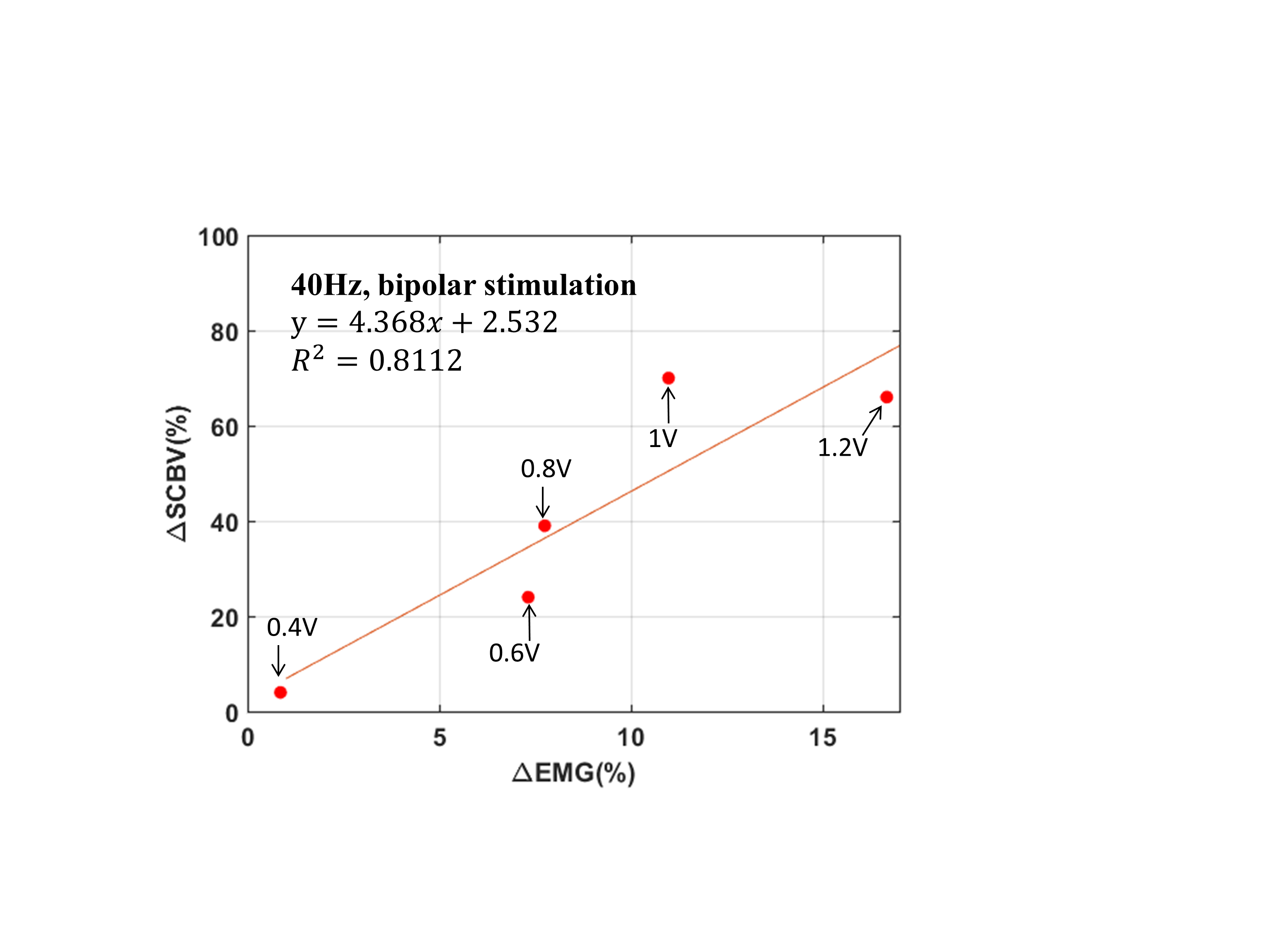

Supplement: Supplementary Figure 1 — Spinal cord hemodynamic response and EMG to a gradient voltage. [file Image_1.TIF]
